# Environmental enteric dysfunction: a review of potential mechanisms, consequences and management strategies

**DOI:** 10.1186/s12916-019-1417-3

**Published:** 2019-11-25

**Authors:** Kirkby D. Tickell, Hannah E. Atlas, Judd L. Walson

**Affiliations:** 1 Department of Global Health, University of Washington, 325 9th Avenue (Box 359931), Seattle, WA 98104 USA; 2 Department of Epidemiology, University of Washington, 1959 NE Pacific Street, Health Sciences Bldg, F-262, Box 357236, Seattle, WA 98195 USA; 3 The Childhood Acute Illness and Nutrition Network (CHAIN), Nairobi, Kenya; 4 Department of Allergy and Infectious Disease, University of Washington, 325 9th Avenue (Box 359931), Seattle, WA 98104 USA; 5 Department of Pediatrics, University of Washington, 325 9th Avenue (Box 359931), Seattle, WA 98104 USA

**Keywords:** Environmental enteric dysfunction, Enteric dysfunction, Childhood malnutrition, Acute malnutrition, Stunting

## Abstract

**Background:**

Environmental enteric dysfunction (EED) is an acquired enteropathy of the small intestine, characterized by enteric inflammation, villus blunting and decreased crypt-to-villus ratio. EED has been associated with poor outcomes, including chronic malnutrition (stunting), wasting and reduced vaccine efficacy among children living in low-resource settings. As a result, EED may be a valuable interventional target for programs aiming to reduce childhood morbidity in low and middle-income countries.

**Main text:**

Several highly plausible mechanisms link the proposed pathophysiology underlying EED to adverse outcomes, but causal attribution of these pathways has proved challenging. We provide an overview of recent studies evaluating the causes and consequences of EED. These include studies of the role of subclinical enteric infection as a primary cause of EED, and efforts to understand how EED-associated systemic inflammation and malabsorption may result in long-term morbidity. Finally, we outline recently completed and upcoming clinical trials that test novel interventions to prevent or treat this highly prevalent condition.

**Conclusions:**

Significant strides have been made in linking environmental exposure to enteric pathogens and toxins with EED, and in understanding the multifactorial mechanisms underlying this complex condition. Further insights may come from several ongoing and upcoming interventional studies trialing a variety of novel management strategies.

## Background

Environmental enteric dysfunction was first described among adult Peace Corps volunteers returning from deployment to low and middle-income countries (LMIC) in the 1960s who presented with unexplained, persistent weight loss. Despite no specific, clearly identifiable infectious etiology, biopsies of intestinal tissue in these individuals demonstrated morphological changes suggestive of chronic enteric infection [1, 2]. The symptoms of these volunteers usually resolved within several months of returning to the USA [3], further supporting the link between these histological changes and recurrent exposure to pathogens in areas of poor sanitation and hygiene. The hypothesized environmental etiology and histopathological evidence of enteropathy has led some investigators to call this condition ‘environmental enteropathy’ [4, 5]. However, evidence of decreased enteric absorptive capacity and barrier function associated with this enteropathy has led some investigators to shift from ‘environmental enteropathy’ to the term ‘environmental enteric dysfunction’ (EED) [6, 7]. In support of this hypothesis, abnormal biomarkers suggestive of EED have been found to be highly prevalent among children in multiple low-resource settings. These biomarkers have been associated with linear and ponderal growth deficits [8–11]. Given these findings, and the clear public health importance of malnutrition and growth failure, EED has become an important potential target for intervention.

Several environmental and nutritional factors can cause enteropathy in LMIC settings, including specific micronutrient deficiencies, diarrheal disease, and chronic infections such as HIV [12]. The coarse histopathology of these conditions is similar, but the etiology of EED, and the mechanisms that link it to negative outcomes, are thought to be distinct. Unfortunately, it has been challenging to definitively establish the causes and consequences of EED – in part because the condition lacks a universally accepted case definition, and there are no universally accepted diagnostic tests or set of diagnostic criteria for EED [13]. As a result, accurately estimating the distribution, burden and underlying mechanisms driving EED is difficult.

The geographic distribution of EED suggests that the syndrome is most prevalent in areas of poor access to improved water and sanitation. In addition, biomarkers of EED have been strongly associated with storage of fecal matter near households and unimproved water sources in LMICs [14]. These findings suggest that EED is the result of exposure to environmental contamination. Molecular detection of enteric pathogens has confirmed that children living in LMIC settings harbor concurrent and consecutive enteric pathogens for much of their early childhood [15–17]. The Etiology, Risk Factors and Interactions of Enteric Infections and Malnutrition and the Consequences for Child Health and Development (MAL-ED) study, a large multi-country birth cohort designed to evaluate causes of childhood stunting, reported that children with identified enteric pathogens exhibited increased enteric inflammation and decreased linear growth, even in the absence of diarrhea [18]. Several specific pathogens, including *Campylobacter, Shigella, Yersinia* and *Giardia*, appear to have stronger associations with enteric inflammation and linear growth failure [19]. Many of these pathogens primarily affect children over 6 months of age – the age at which exclusive breastfeeding often ends, and the prevalence of stunting begins to increase rapidly [20]. This timing may be a clue as to the age-specific window in which EED drives growth failure, and could represent an optimal period for EED focused interventions.

## Mechanisms and consequences

Five highly interdependent mechanisms may link EED to poor health outcomes: 1) increased intestinal permeability with translocation of bacteria or antigens, 2) chronic intestinal inflammation without translocation, 3) malabsorption, 4) hormonal disruption, and 5) microbiome disruption.

The healthy intestine serves as a physical barrier between the gut lumen and the systemic circulation. In EED, disruption of the gut architecture, with breakdown of tight junctions between cells, creates a permeable intestine that can allow bacteria or bacterial products to translocate into the systemic circulation [18]. This may lead to subsequent immune activation and a systemic inflammatory state, with associated downstream health effects. For example, acute phase proteins induced by translocation have been shown to inhibit insulin-like growth factor 1 (IGF-1), and lead to growth hormone resistance [21]. This may suppress linear growth [22], affect cognitive development, and detrimentally affect immune responses to pathogen challenge [23, 24]. In addition, the indoleamine-2,3-dioxygenase 1 pathway serves as a marker of systemic inflammation, and has also been associated with reduced polio vaccine efficacy [25]. However, it is important to note that chronic systemic inflammation can occur in the absence of translocation. To date, few studies have found direct evidence linking systemic inflammation to enteric translocation [18, 24].

Malabsorption also potentially links EED to negative outcomes. EED substantially damages intestinal structure, including causing shortened and blunted villi and crypt hyperplasia, which lead to a loss of absorptive intestinal surface area [7, 26]. Deficits in the absorption of essential nutrients arising from this loss of surface area could result in metabolic pathway derangement, or simply a mismatch between the availability and consumption of micronutrients and macronutrients. However, other models of poor absorptive capacity, such as that observed in children with inflammatory bowel disease, suggest that even when substantial sections of small intestine are resected, these children often maintain relatively normal gut function [27]. Interestingly, while the MAL-ED study reported a strong association between the presence of systemic inflammation and linear growth, ponderal growth was less affected by inflammation. It may be that malabsorption is a more critical driver of weight loss and wasting than systemic inflammation [18].

EED may also be associated with enteric microbiome dysbiosis. EED has been associated with changes to the microbiome, as loss of gut surface area and profound enteric inflammation alter the ecological niches that support certain bacterial taxa. The microbiome contributes to multiple homeostatic mechanisms, and undernourished children have been shown to have both a reduced diversity in the enteric microbiome, and a decrease in specific taxa associated with healthy childhood growth [28, 29]. Administration of these specific growth-promoting or growth-suppressing taxa have also been shown to reproduce or ameliorate growth failure in mice [28]. A healthy microbiome protects against pathogen colonization and invasion, including with *Shigella* and other diarrheagenic pathogens, and may also protect against subclinical pathogen colonization and EED [29]. The microbiome also aids the body in liberating calories from ingested food; EED-associated dysbiosis may exacerbate nutrient deficits [29]. Finally, the microbiome is a key regulator of hormonal responses to feeding and fasting. These hormonal changes have been linked to EED, including reductions in IGF-1 and fibroblast growth factor 21 [21, 30].

## Identification

EED is most clearly diagnosed by observing well-described alterations in the histology of the small intestine. As a result, upper gastrointestinal endoscopy with biopsy is the current gold standard for diagnosis. However, access to endoscopy is severely limited in most EED endemic settings, and – even where available – concerns about safety limit its utility for routine diagnosis. Although new technologies, such as capsule endoscopy with biopsy, may soon be available [31, 32], it is unlikely that endoscopy-based diagnostics will be implemented at scale.

**Fig. 1:**
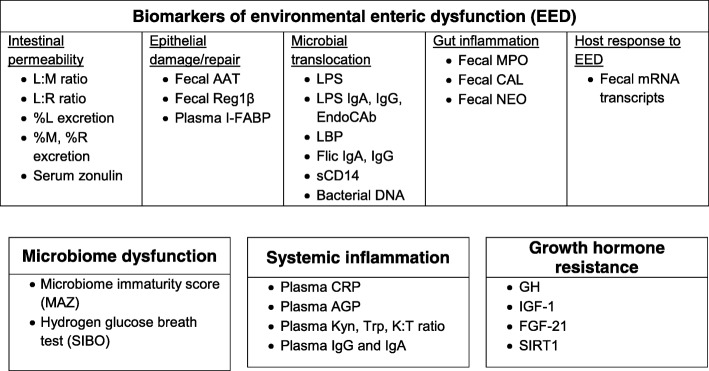
Biomarkers of environmental enteric dysfunction (EED), microbiome dysfunction, systemic inflammation and growth hormone resistance. Adapted from McGrath (2017) [17]. Abbreviations: AAT, α-1-antitrypsin; AGP, α-1 acid glycoprotein; CAL, calprotectin; CRP, C-reactive protein; EndoCAb, anti-endotoxin core antibody; FGF-21, fibroblast growth factor 21; Flic, flagellin; GH, Growth hormone; I-FABP, intestinal fatty acid binding protein; IgA, immunoglobulin A; IgG, immunoglobulin G; IGF-1, insulin-like growth factor 1; Kyn, kynurenine; K:T, kynurenine:tryptophan ratio; LPS, lipopolysaccharides; L:M, lactulose:mannitol; L:R, lactulose:rhamnose; MAZ, microbiota-for-age Z score; MPO, myeloperoxidase; NEO, neopterin; Reg1β, regenerating protein 1β; SIBO, small intestinal bacterial overgrowth; SIRT1, Sirtuin 1; Trp, tryptophan

Therefore, a variety of biomarkers targeting proposed pathways have been evaluated as EED diagnostics (Fig. 1). These biomarkers are less invasive than endoscopy, and are drawn from a variety of body compartments, including urine, stool and blood, but there are no widely accepted diagnostic criteria that utilize these tests. The dual-sugar permeability test has been the most widely implemented of these surrogate markers. This is based on the premise that a healthy intestine will absorb small sugars (mannitol or rhamnose), while keeping large sugars (lactulose) from entering the systemic circulation, thus delivering an active assessment of gut function [33, 34]. In EED, tight junctions between the intestinal cells are disrupted, allowing the larger sugars to pass into the body’s circulation. As a result, both types of sugar are excreted by the kidney, and the ratio of the two sugars is indicative of the degree of permeability in the intestine. The lactulose:mannitol ratio (L:M) and lactulose:rhamnose ratio (L:R) have been shown to be associated with linear growth faltering [8]. However, the test can take 2–5 hours, and requires considerable experience to implement. This procedure can also yield inconsistent results, perhaps owing to the lack of standardized procedures and reporting [33].

Fecal and plasma biomarkers of inflammation are also available [9, 10, 25, 35, 36]. However, no single biomarker or collection of biomarkers has been systematically validated across geographic settings and populations [24, 37]. Several ongoing studies are attempting to correlate these biomarkers with histology using selective endoscopy in specific populations [38, 39].

## Prevention and management

Effective interventions to prevent or treat EED in low-resource settings are limited. Given the apparent association between environmental exposures and EED, efforts to minimize environmental contamination through water, sanitation and hygiene (WASH) interventions have been a focus of several large interventional trials. Two recently completed, highly rigorous, cluster-randomized controlled trials estimated the efficacy of WASH interventions in reducing childhood diarrhea, limiting EED and improving childhood growth. A significant reduction in diarrhea incidence was observed among children receiving WASH interventions in Bangladesh, but this finding was not replicated in Kenya or Zimbabwe. Furthermore, WASH interventions were not associated with improved linear growth in any of these studies [40–42]. It is likely that community-wide improvements in water and sanitation infrastructure would lessen the burden of EED, but these studies suggest that individual or household-level WASH interventions may not provide sufficient protection from environmental contamination to prevent or ameliorate EED.

Treatment of documented EED may be a more feasible approach given the ubiquitous environmental contamination in many LMIC settings. Several recently completed or ongoing studies are evaluating approaches to reducing the impact of EED in LMIC settings. We identified 16 ongoing or completed interventional studies (Table 1) of interventions for EED, which we group into three strategies: anti-inflammatory drugs, antimicrobial interventions and dietary supplements.

**Table 1:** Interventional studies testing management strategies for EED, or using interventions to better understand EED

Study title	Study population	Target sample size	Intervention arm(s)	Control arm(s)	Primary outcome(s)
**Complete**					
Lactoferrin and lysozyme supplementation for environmental enteric dysfunction	Children 12–25 months old, Malawi	235	Lactoferrin and lysozyme, 16 weeks	Placebo	L:M at 8 and 16 weeks
The impact of legumes vs corn-soy flour on environmental enteric dysfunction in rural Malawian children 1–3 year olds	Children 12–35 months old, Malawi	337	Cowpeas complementary food, 12 months Common bean complementary food, 12 months	Corn-soy flour	L:M at 3, 6, and 12 months
The impact of legumes vs corn-soy flour on environmental enteric dysfunction in rural Malawian children 6–11 months	Children 6–11 months old, Malawi	312	Cowpeas complementary food, 6 months Common bean complementary food, 6 months	Corn-soy flour	L:M at 3 and 6 months
Intervention and mechanisms of alanyl-glutamine for inflammation, nutrition, and enteropathy (IMAGINE)	Children 2 months to 5 years old, ≤ − 1 for: HAZ WAZ, or WHZ, Brazil	112	Alanyl-glutamine, 10 days	Glycine for 10 days	L:M on D 1, 10–13, 30–37
Mesalazine in the initial management of severely acutely malnourished children with environmental enteric dysfunction	Children 12–60 months old with mid upper arm circumference < 11.5 cm or bilateral pedal edema, Kenya	44	Mesalazine, 28 days	Placebo	Safety and acceptability at 28 days
**Ongoing**					
Azithromycin to prevent post-discharge morbidity and mortality in Kenyan children (Toto Bora)	Children 1–59 months old discharged from hospital, Kenya	1400	Azithromycin, 5 days	Placebo	Death or readmission with 6 months
Pilot study of PTM202 for the treatment of environmental enteric dysfunction (EED)	Children 6–9 months old WAZ −1 to − 3, Bangladesh	200	PTM202, 30 days	Micronutrient sprinkles	EED biomarker composite score (REG1B, MPO, L:M, sCD14, CRP) at 4 months
Clinical effectiveness trial of PTM1001 to reduce stunting and ameliorate environmental enteric dysfunction in Malawian infants	Healthy children 10 months old, Malawi	250	Egg powder + bovine colostrum + multiple micronutrient sprinkle powder, 12 weeks	Corn-soya blend + multiple micronutrient sprinkle powder	Linear growth at 12 weeks
Protein Plus: improving infant growth through diet and enteric health (JiVitA-6)	Children 3–6 months old born to women enrolled in ongoing community trial, Bangladesh	3180	Azithromycin Azithromycin, protein Azithromycin, isocaloric supplement Azithromycin, daily egg provided to infants 6–12 months 3 days	Placebo Placebo, protein supplement Placebo, isocaloric supplement, Placebo, egg provided daily to infants 6–12 months old	HAZ at 12 months
Effects of EED on Zinc absorption and retention in children from a standard dose (ZEED1)	Children with and without EED (defined by L:M), Bangladesh	40	Zinc sulfate supplement, 0.5 mg of 13C10-retinyl-acetate, 1 day	–	Zinc absorption Endogenous fecal zinc Vitamin A absorption
Effects of EED on Zinc absorption and retention in children from a MNP (ZEED2)	Children 18–24 months old with LAZ−1.5 to −3.0 and hemoglobin ≥8, Bangladesh	80	Micronutrients + 15 mg zinc Micronutrients + 10 mg zinc Micronutrient + 5 mg zinc 1 day	Micronutrient without zinc	Total daily absorbed zinc
Early life interventions for childhood growth and development in Tanzania (ELICIT)	Children ≤14 days old, Tanzania	1188	Nicotinamide, azithromycin, nitazoxanide Placebo, azithromycin, nitazoxanide Nicotinamide, placebo, placebo	Azithromycin, placebo, Nitazoxanide, placebo	HAZ at 18 months
Safety, acceptability, and feasibility of Enterade® (SAFE)	Children 12–24 months old, HAZ − 3 to − 1, Kenya	66	Enterade, 14 days	Placebo	Frequency of severe adverse events Volume of daily consumption
Therapeutic approaches to malnutrition enteropathy (TAME)	Children 6–59 months old, hospitalized with severe acute malnutrition and clinically stable, Zambia	235	Colostrum GInNAC Teduglutide Budenoside 14 days	Standard of care WHO guidelines of severe acute malnutrition	EED composite score at 18 days
Study of environmental enteropathy and malnutrition	Children 0–6 months old, WHZ < −2, Pakistan Children 0–6 months old, WHZ > 0, Pakistan Healthy children < 3 years old, USA Children < 6 years old, newly diagnosed celiac disease, USA Children < 10 years old with newly diagnosed Crohn’s disease, USA	500	Ready-to-use supplementary foods (AchaMum), 60–90 days	Educational program focused on breastfeeding and complementary feeding No intervention: US children with celiac disease, Crohn’s disease, and healthy age-matched US children	Nutritional status at 3–6 months and 9 months Association biomarkers with EED at 3–6 months Association of biomarkers with EED at 9 months Association biomarkers at time of endoscopy and biopsy Upper gastrointestinal tissue biopsy and multiomic validation of EED biomarkers
Environmental enteropathy in Zambia: biomarkers defined by pathogenesis	Children < 18 months old, HAZ < − 2, Zambia	400	HEPS (corn-soya blend) + daily egg + micronutrient sprinkles	–	UGI endoscopy with validation of EED biomarkers

*Abbreviations: EED* Environmental enteric dysfunction, *HAZ* Height-for-age z-score, *L:M* Lactulose-to-mannitol ratio, *WAZ* Weight-for-age z-score, *WHZ* Weight-for-height z-score

Therapeutics developed for inflammatory bowel disease may have a role in treating EED, as these conditions share features of enteric inflammation, loss of intestinal architecture and systemic inflammation. However, many of these medications have adverse side effect profiles, and may not be acceptable for use in young children in these settings. The safety of mesalazine use has been evaluated in malnourished children, and no detectable increase in adverse events was reported [43]. In addition, a pilot trial of budesonide is ongoing in Zambia and Zimbabwe.

Given the hypothesized role of enteric infection in the pathogenesis of EED, several studies are attempting selective gut decontamination with antimicrobials [44, 45]. Antibiotics may promote linear growth [46], and recent trials of biannual azithromycin mass drug administration (MDA) have demonstrated a reductions in all cause childhood mortality [47]. Antibiotics may also provide a pathogen-free window for the enteric system to recover following insult. Although there are clearly concerns related to the emergence of antimicrobial resistance, antibiotics are already widely utilized in these settings. For example, children under the age of two included in the MAL-ED study received an average of five courses of antibiotics per year [48]. In addition, determining whether antibiotics play an important role in the management of EED would permit more clear guidelines for antibiotic use, which has been shown to result in decreased misuse of antibiotics overall [49]. There is also considerable interest in the use of probiotics or prebiotics for the treatment of EED, but to date only a single study has evaluated the administration of a probiotic (*Lactobacillus* GG) and it found no effect on measures of EED [50].

We identified 10 trials evaluating dietary supplements for EED. These can be divided into protein supplementation, micronutrient supplementation, probiotics, and naturally occurring novel supplements. Five studies combine dietary supplementation with additional protein or other complementary foods, both of which have been shown to increase childhood growth [51–54]. Extensive evidence also exists regarding the role of many micronutrients in promoting childhood growth, largely demonstrating no association or clinically insignificant effects when supplements are provided [55, 56]. The prospect of treating or preventing EED with micronutrients that modulate the immune response, for example with the use of nicotinamide, is the subject of current evaluation [45].

Four studies of novel dietary supplements were identified, including breast milk derivatives and alanyl-glutamine. Identifying components of breast milk that protect children from diarrhea in the first six-months of life may offer the opportunity to supplement beyond this period and provide extended protection to older children. A recently published study of bovine and recombinant human lactoferrin and lysozyme reported no significant effect on lactulose excretion [53]. However, the intervention did reduce the incidence of malnutrition and hospitalization in the included children. In addition, two studies are currently piloting the use of bovine colostrum derivates [57, 58], one in combination with N-acetyl glucosamine, an amino acid thought to reduce enteric inflammation [59].

In addition to identifying effective interventions, considering the optimal delivery strategy for these interventions are also needed. Given the highly prevalent nature of EED in many settings, empiric treatment of entire populations through MDA may be a viable delivery mechanism. MDA is a highly equitable delivery platform [60], which may help to ensure that the highest risk children are effectively captured for intervention. However, MDA requires that interventions are inexpensive and safe, which limits its ability to support many of the therapeutics currently being evaluated. Screen-and-treat approaches are an alternative to MDA, but this approach would be complicated by the lack of a universally accepted case definition for EED, or an easily administered diagnostic [13]. Screen-and-treat policies are also relatively more expensive. Interventions could also be administered to a targeted group of high-risk individuals, such as severely malnourished children or children presenting to medical facilities with an acute illness. Given that mortality is concentrated in these populations, this strategy may reach the greatest number of children with capacity to benefit, while limiting the cost and drug exposure of a less targeted approach [61]. However, achieving high coverage in selected populations can be challenging. Community-Based Management of Acute Malnutrition programs are highly cost-effective [61–63], but only reach 17% of acutely malnourished children [64], and only 44% of children with diarrhea currently receive oral rehydration solution [65]. These data suggest that the malnutrition management and medical care platforms in LMIC settings would also benefit from investment and scale-up if they are to be an effective EED treatment platform.

## Conclusion

Understanding and addressing the etiology of childhood wasting and stunting, and the consequences of these syndromes, remains a global public health priority. Significant strides have been made in linking environmental exposure to enteric pathogens and toxins with EED, and in understanding the multifactorial mechanisms underlying this complex condition. Further insights may come from several ongoing and upcoming interventional studies, which propose several novel management strategies. However, the potential of these interventions to reduce the global burden of morbidity associated with EED will be limited by the strength of the delivery platforms they target. It is vital that novel intervention development is accompanied by investment in healthcare platforms that can be leveraged to deliver effective managements.

## Data Availability

Not applicable.

## Abbreviations

EED: Environmental enteric dysfunction
IGF-1: Insulin-like growth factor 1
L:M: Lactulose:mannitol ratio
L:R: Lactulose:rhamnose ratio
LMIC: Low and middle-income countries
MAL-ED: Etiology, Risk Factors and Interactions of Enteric Infections and Malnutrition and the Consequences for Child Health and Development
MDA: Mass drug administration
WASH: Water, sanitation and hygiene

## References

[1] Keusch GTPA, Troncale FJ (1972). Subclinical malabsorption in Thailand. II. Intestinal absorption in American military and peace corps personnel. Am J Clin Nutr.

[2] Lindenbaum JKT, Sprinz H (1966). Malabsorption and jejunitis in American peace corps volunteers in Pakistan. Ann Intern Med.

[3] Lindenbaum JGC, Kent TH (1971). Recovery of small-intestinal structure and function after residence in the tropics. I. Studies in peace corps volunteers. Ann Intern Med.

[4] Korpe PS, Petri WA (2012). Jr..Environmental enteropathy: critical implications of a poorly understood condition. Trends Mol Med.

[5] Louis-Auguste J, Kelly P (2017). Tropical enteropathies. Curr Gastroenterol Rep.

[6] Keusch GT, Rosenberg IH, Denno DM, Duggan C, Guerrant RL, Lavery JV (2013). Implications of acquired environmental enteric dysfunction for growth and stunting in infants and children living in low- and middle-income countries. Food Nutr Bull.

[7] Keusch GT, Denno DM, Black RE, Duggan C, Guerrant RL, Lavery JV (2014). Environmental enteric dysfunction: pathogenesis, diagnosis, and clinical consequences. Clin Infect Dis.

[8] Campbell RK, Schulze KJ, Shaikh S, Mehra S, Ali H, Wu L (2017). Biomarkers of environmental enteric dysfunction among children in rural Bangladesh. J Pediatr Gastroenterol Nutr.

[9] Kosek M, Haque R, Lima A, Babji S, Shrestha S, Qureshi S (2013). Fecal markers of intestinal inflammation and permeability associated with the subsequent acquisition of linear growth deficits in infants. Am J Trop Med Hyg..

[10] Guerrant RL, Leite AM, Pinkerton R, Medeiros PH, Cavalcante PA, DeBoer M (2016). Biomarkers of environmental enteropathy, inflammation, stunting, and impaired growth in children in Northeast Brazil. PLoS One.

[11] McCormick BJJ, Lee GO, Seidman JC, Haque R, Mondal D, Quetz J (2017). Dynamics and trends in fecal biomarkers of gut function in children from 1–24 months in the MAL-ED study. Am J Trop Med Hyg..

[12] Owino V, Ahmed T, Freemark M, Kelly P, Loy A, Manary M, et al. Environmental enteric dysfunction and growth failure/stunting in global child health. Pediatrics. 2016;138(6):e20160641. 10.1542/peds.2016-0641 27940670

[13] Denno DM, Tarr PI, Nataro JP (2017). Environmental enteric dysfunction: a case definition for intervention trials. Am J Trop Med Hyg..

[14] Exum NG, Lee GO, Olortegui MP, Yori PP, Salas MS, Trigoso DR (2018). A longitudinal study of household water, sanitation, and hygiene characteristics and environmental enteropathy markers in children less than 24 months in Iquitos, Peru. Am J Trop Med Hyg..

[15] Liu J, Gratz J, Amour C, Nshama R, Walongo T, Maro A (2016). Optimization of quantitative PCR methods for enteropathogen detection. PLoS One.

[16] Liu J, Platts-Mills JA, Juma J, Kabir F, Nkeze J, Okoi C (2016). Use of quantitative molecular diagnostic methods to identify causes of diarrhoea in children: a reanalysis of the GEMS case-control study. Lancet..

[17] Platts-Mills JA, Liu J, Rogawski ET, Kabir F, Lertsethtakarn P, Siguas M (2018). Use of quantitative molecular diagnostic methods to assess the aetiology, burden, and clinical characteristics of diarrhoea in children in low-resource settings: a reanalysis of the MAL-ED cohort study. Lancet Glob Health.

[18] Kosek MN, Network Investigators MAL-ED (2017). Causal pathways from enteropathogens to environmental enteropathy: findings from the MAL-ED birth cohort study. EBioMedicine..

[19] Rogawski ET, Liu J, Platts-Mills JA, Kabir F, Lertsethtakarn P, Siguas M (2018). Use of quantitative molecular diagnostic methods to investigate the effect of enteropathogen infections on linear growth in children in low-resource settings: longitudinal analysis of results from the MAL-ED cohort study. Lancet Glob Health.

[20] MAL-ED Network Investigators (2017). Childhood stunting in relation to the pre- and postnatal environment during the first 2 years of life: the MAL-ED longitudinal birth cohort study. PLoS Med.

[21] McGrath CJ, Arndt MB, Walson JL (2017). Biomarkers to stratify risk groups among children with malnutrition in resource-limited settings and to monitor response to intervention. Horm Res Paediatr.

[22] Prendergast AJ, Rukobo S, Chasekwa B, Mutasa K, Ntozini R, Mbuya MN (2014). Stunting is characterized by chronic inflammation in Zimbabwean infants. PLoS One.

[23] Rytter MJ, Kolte L, Briend A, Friis H, Christensen VB (2014). The immune system in children with malnutrition--a systematic review. PLoS One.

[24] Harper KM, Mutasa M, Prendergast AJ, Humphrey J, Manges AR (2018). Environmental enteric dysfunction pathways and child stunting: a systematic review. PLoS Negl Trop Dis.

[25] Kosek MN, Mduma E, Kosek PS, Lee GO, Svensen E, Pan WKY (2016). Plasma tryptophan and the kynurenine-tryptophan ratio are associated with the acquisition of statural growth deficits and oral vaccine underperformance in populations with environmental enteropathy. Am J Trop Med Hyg..

[26] Syed S, Ali A, Duggan C (2016). Environmental enteric dysfunction in children. J Pediatr Gastroenterol Nutr.

[27] Guillen B, Atherton NS. Short bowel syndrome. In: StatPearls. Treasure Island, FL: StatPearls; 2019. https://www.ncbi.nlm.nih.gov/books/NBK536935/. Accessed 04 Feb 2019.

[28] Blanton LV, Charbonneau MR, Salih T, Barratt MJ, Venkatesh S, Ilkaveya O, et al. Gut bacteria that prevent growth impairments transmitted by microbiota from malnourished children. Science. 2016;351(6275):aad3311. 10.1126/science.aad3311 PMC4787260 26912898

[29] Nataro JP, Guerrant RL (2017). Chronic consequences on human health induced by microbial pathogens: growth faltering among children in developing countries. Vaccine..

[30] Arndt MB, Richardson BA, Mahfuz M, Ahmed T, Haque R, Gazi MA (2019). Plasma fibroblast growth factor 21 is associated with subsequent growth in a cohort of underweight children in Bangladesh. Curr Dev Nutr.

[31] Gora MJ, Sauk JS, Carruth RW, Gallagher KA, Suter MJ, Nishioka NS (2013). Tethered capsule endomicroscopy enables less invasive imaging of gastrointestinal tract microstructure. Nat Med.

[32] Thompson AJ, Hughes M, Anastasova S, Conklin LS, Thomas T, Leggett C (2017). Position paper: the potential role of optical biopsy in the study and diagnosis of environmental enteric dysfunction. Nat Rev Gastroenterol Hepatol.

[33] Denno DM, VanBuskirk K, Nelson ZC, Musser CA, Hay Burgess DC, Tarr PI (2014). Use of the lactulose to mannitol ratio to evaluate childhood environmental enteric dysfunction: a systematic review. Clin Infect Dis.

[34] Faubion WA, Camilleri M, Murray JA, Kelly P, Amadi B, Kosek MN (2016). Improving the detection of environmental enteric dysfunction: a lactulose, rhamnose assay of intestinal permeability in children aged under 5 years exposed to poor sanitation and hygiene. BMJ Glob Health.

[35] Arndt MB, Richardson BA, Ahmed T, Mahfuz M, Haque R, John-Stewart GC (2016). Fecal markers of environmental enteropathy and subsequent growth in Bangladeshi children. Am J Trop Med Hyg.

[36] Iqbal NT, Sadiq K, Syed S, Akhund T, Umrani F, Ahmed S (2018). Promising biomarkers of environmental enteric dysfunction: a prospective cohort study in Pakistani children. Sci Rep.

[37] Denno DM, VanBuskirk KM, Nelson ZC, Musser CA, Tarr PI (2016). Environmental enteric dysfunction: advancing current knowledge.

[38] Mahfuz M, Das S, Mazumder RN, Masudur Rahman M, Haque R, Bhuiyan MMR (2017). Bangladesh environmental enteric dysfunction (BEED) study: protocol for a community-based intervention study to validate non-invasive biomarkers of environmental enteric dysfunction. BMJ Open.

[39] ClinicalTrials.gov. Identifier NCT03588013, Study of Environmental Enteropathy and Malnutrition in Pakistan (SEEM); 2018. https://clinicaltrials.gov/ct2/show/NCT03588013?term=NCT03588013&rank=1. Accessed 04 Feb 2019.

[40] Luby SP, Rahman M, Arnold BF, Unicomb L, Ashraf S, Winch PJ (2018). Effects of water quality, sanitation, handwashing, and nutritional interventions on diarrhoea and child growth in rural Bangladesh: a cluster randomised controlled trial. Lancet Glob Health.

[41] Null C, Stewart CP, Pickering AJ, Dentz HN, Arnold BF, Arnold CD (2018). Effects of water quality, sanitation, handwashing, and nutritional interventions on diarrhoea and child growth in rural Kenya: a cluster-randomised controlled trial. Lancet Glob Health.

[42] Humphrey JH, Mbuya MNN, Ntozini R, Moulton LH, Stoltzfus RJ, Tavengwa NV (2019). Independent and combined effects of improved water, sanitation, and hygiene, and improved complementary feeding, on child stunting and anaemia in rural Zimbabwe: a cluster-randomised trial. Lancet Glob Health.

[43] Jones KD, Hünten-Kirsch B, Laving AM, Munyi CW, Ngari M, Mikusa J (2014). Mesalazine in the initial management of severely acutely malnourished children with environmental enteric dysfunction: a pilot randomized controlled trial. BMC Med.

[44] Pavlinac PB, Singa BO, John-Stewart GC, Richardson BA, Brander RL, McGrath CJ (2017). Azithromycin to prevent post-discharge morbidity and mortality in Kenyan children: a protocol for a randomised, double-blind, placebo-controlled trial (the Toto bora trial). BMJ Open.

[45] DeBoer MD, Platts-Mills JA, Scharf RJ, McDermid JM, Wanjuhi AW, Gratz J (2018). Early life interventions for childhood growth and development in Tanzania (ELICIT): a protocol for a randomised factorial, double-blind, placebo-controlled trial of azithromycin, nitazoxanide and nicotinamide. BMJ Open.

[46] Gough EK, Moodie EE, Prendergast AJ, Johnson SM, Humphrey JH, Stoltzfus RJ (2014). The impact of antibiotics on growth in children in low and middle income countries: systematic review and meta-analysis of randomised controlled trials. BMJ.

[47] Keenan JD, Bailey RL, West SK, Arzika AM, Hart J, Weaver J (2018). Azithromycin to reduce childhood mortality in sub-Saharan Africa. N Engl J Med.

[48] Rogawski ET, Platts-Mills JA, Seidman JC, John S, Mahfuz M, Ulak M (2017). Use of antibiotics in children younger than two years in eight countries: a prospective cohort study. Bull World Health Organ.

[49] Qazi SA, Rehman GN, Khan MA (1996). Standard management of acute respiratory infections in a children’s hospital in Pakistan: impact on antibiotic use and case fatality. Bull World Health Organ.

[50] Galpin L, Manary MJ, Fleming K, Ou CN, Ashorn P, Shulman RJ (2005). Effect of Lactobacillus GG on intestinal integrity in Malawian children at risk of tropical enteropathy. Am J Clin Nutr.

[51] Stephenson KB, Agapova SE, Divala O, Kaimila Y, Maleta KM, Thakwalakwa C (2017). Complementary feeding with cowpea reduces growth faltering in rural Malawian infants: a blind, randomized controlled clinical trial. Am J Clin Nutr.

[52] Agapova SE, Stephenson KB, Divala O, Kaimila Y, Maleta KM, Thakwalakwa C (2018). Additional common bean in the diet of Malawian children does not affect linear growth, but reduces intestinal permeability. J Nutr.

[53] Cheng WD, Wold KJ, Bollinger LB, Ordiz MI, Shulman RJ, Maleta KM (2019). Supplementation with lactoferrin and lysozyme ameliorates environmental enteric dysfunction: a double-blind, randomized, placebo-controlled trial. Am J Gastroenterol.

[54] Desai A, Smith LE, Mbuya MN, Chigumira A, Fundira D, Tavengwa NV (2015). The SHINE trial infant feeding intervention: pilot study of effects on maternal learning and infant diet quality in rural Zimbabwe. Clin Infect Dis.

[55] De-Regil LM, Suchdev PS, Vist GE, Walleser S, Peña-Rosas JP. Home fortification of foods with multiple micronutrient powders for health and nutrition in children under two years of age. Cochrane Database Syst Rev. 2011;(9):CD008959. 10.1002/14651858.CD008959.pub2 21901727

[56] Mayo-Wilson E, Junior JA, Imdad A, Dean S, Chan XHS, Chan ES, et al. Zinc supplementation for preventing mortality, morbidity, and growth failure in children aged 6 months to 12 years of age. Cochrane Database Syst Rev. 2014;(5):CD009384. 10.1002/14651858.CD009384.pub2 24826920

[57] ClinicalTrials.gov. Identifier NCT03716115, Therapeutic Approaches to Malnutrition Enteropathy (TAME); 2018. https://clinicaltrials.gov/ct2/show/NCT03716115?term=Therapeutic+Approaches+to+Malnutrition+Enteropathy+%28TAME%29&rank=1. Accessed 04 May 2019.

[58] ClinicalTrials.gov. Identifier NCT03263871, pilot study of PTM202 for the treatment of environmental enteric dysfunction (EED). https://clinicaltrials.gov/ct2/show/NCT03263871?term=Pilot+Study+of+PTM202+for+the+Treatment+of+Environmental+Enteric+Dysfunction+%28EED%29&rank=1. Accessed 04 May 2019.

[59] Salvatore S, Heuschkel R, Tomlin S, Davies SE, Edwards S, Walker-Smith JA (2000). A pilot study of N-acetyl glucosamine, a nutritional substrate for glycosaminoglycan synthesis, in paediatric chronic inflammatory bowel disease. Aliment Pharmacol Ther.

[60] Cohn DA, Kelly MP, Bhandari K, Zoerhoff KL, Batcho WE, Drabo F, et al. Gender equity in mass drug administration for neglected tropical diseases: data from 16 countries. Int Health. 2019;ihz012. 10.1093/inthealth/ihz012. 10.1093/inthealth/ihz012 PMC6748770 30845318

[61] Bhutta ZA, Das JK, Rizvi A, Gaffey MF, Walker N, Horton S (2013). Evidence-based interventions for improvement of maternal and child nutrition: what can be done and at what cost?. Lancet..

[62] Puett C, Sadler K, Alderman H, Coates J, Fiedler JL, Myatt M (2013). Cost-effectiveness of the community-based management of severe acute malnutrition by community health workers in southern Bangladesh. Health Policy Plan.

[63] Horton S, Shekar M, McDonald D, Mahal A, Brooks JK (2010). Scaling up nutrition – what will it cost?.

[64] United Nations Children’s Fund (UNICEF) (2015). Management of severe acute malnutrition in children: working towards results at scale.

[65] United Nations Children’s fund (UNICEF). Diarrhoeal disease. 2018. https://data.unicef.org/topic/child-health/diarrhoeal-disease/. Accessed 04 May 2019.

